# Research with variola virus after smallpox eradication: Development of a mouse model for variola virus infection

**DOI:** 10.1371/journal.ppat.1009911

**Published:** 2021-09-21

**Authors:** Bernard Moss, Geoffrey L. Smith

**Affiliations:** 1 Laboratory of Viral Diseases, National Institute of Allergy and Infectious Diseases, National Institutes of Health, Bethesda, Maryland, United States of America; 2 Department of Pathology, University of Cambridge, Cambridge, United Kingdom; King’s College London, UNITED KINGDOM

**Fig 1:**
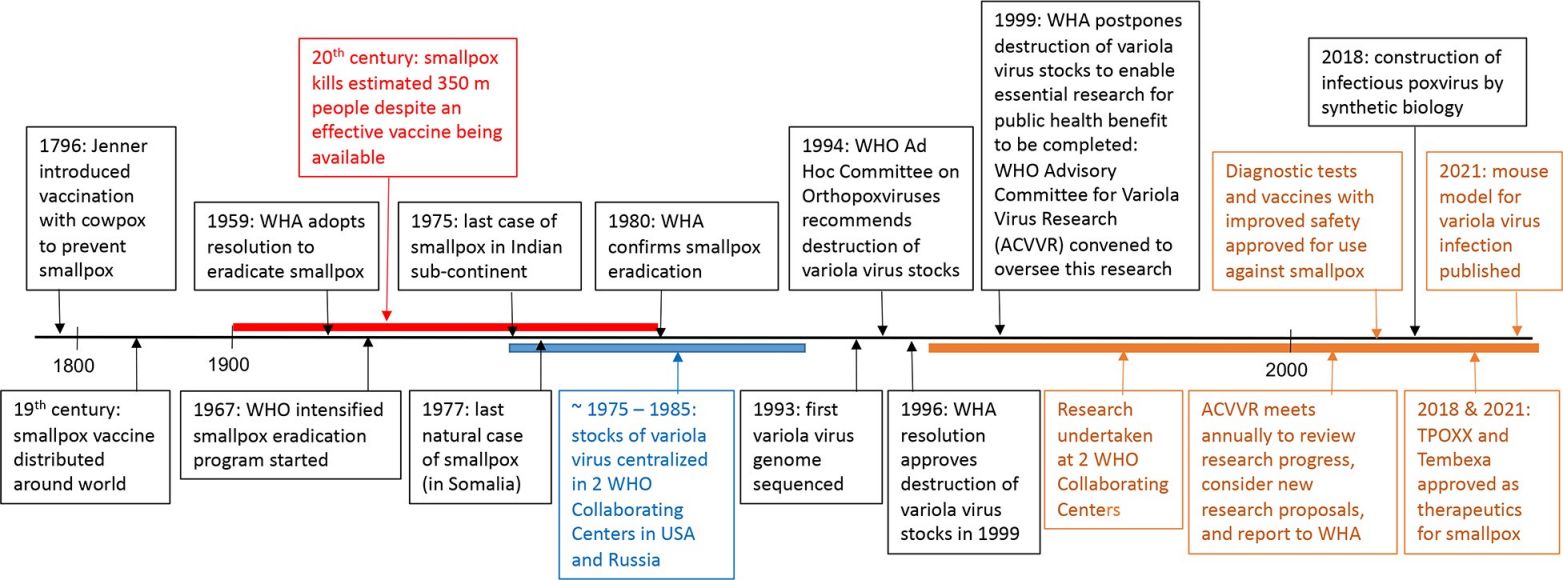
Timeline of significant events.

In this issue of PLOS Pathogens, Hutson and coworkers at the Centers for Disease Control and Prevention (CDC) in Atlanta, USA describe a study with infectious variola virus that was approved by the World Health Organization (WHO) Advisory Committee on Variola Virus Research (ACVVR). Since such research is controversial, the editors felt that readers might appreciate the following background information. Smallpox, a devastating disease caused by variola virus, may have existed for 3,000 or more years. Although the pioneering observations of Benjamin Jesty and Edward Jenner in late 18^th^ century England provided the basis for control of smallpox by inoculation with the cowpox, the disease remained uncontrolled in every continent and virtually every country more than 100 years later (Fig 1). Despite improved methods of immunization with vaccinia virus, in 1967 the disease was still endemic in 33 countries and in that year alone caused an estimated 10–15 million cases, with 2 million deaths, prompting the WHO to renew a world-wide surveillance and vaccination campaign. With the participation of hundreds of thousands of health workers, the spread of variola virus was halted and the last natural case of smallpox occurred in 1977 in Somalia. Smallpox was officially declared eradicated by the WHO Global Commission for the Eradication of Smallpox in 1980. The Global Commission made several recommendations including the retention of variola virus stocks and complete viral genomes exclusively at WHO collaborating centers, which were to be inspected periodically to ensure that storage is secure and that safe operating conditions are maintained. All other laboratories were asked to destroy their variola virus stocks or transfer them to one of the approved laboratories, which were identified as the CDC in Atlanta, USA and the Research Institute of Virus Preparations in Moscow (later moved to Vector in Koltsovo, Russia). The Commission also recommended the establishment of the Committee on Orthopoxvirus Infections, which met annually from 1982 until 1988. The Committee affirmed the importance of selected research on variola virus in the collaborating centers. Thereafter, a WHO Ad Hoc Committee on Orthopoxviruses was established and asked to consider whether the remaining variola virus stocks should be retained or destroyed. This committee met in 1990, 1994, and 1999 and following debate in 1994 recommended that all variola virus stocks held at the Collaborating Centers be destroyed. The World Health Assembly (WHA) endorsed this recommendation in 1996 and a destruction date for June 1999 was set, with the intervening 3 years to allow the completion of any research with infectious variola virus that was essential for public health benefit. However, in 1999, noting that all essential work with variola virus was not yet complete, the WHA reviewed this recommendation and approved a further temporary retention of variola virus stocks so this research could be completed. The objectives of this research were to develop diagnostic tests, antiviral agents and improved vaccines for smallpox. The ACVVR was established in 1999 to oversee this research and has met every subsequent year to review progress towards these objectives, ensure the safe storage and use of variola virus, and to consider further research proposals. In brief, proposals for work with infectious variola virus from the two collaborating centers are submitted to WHO and debated either by the whole ACVVR or a technical sub-committee that includes poxvirus experts. Permission is either refused or given for a defined period and purpose. Results of research are submitted at the ACVVR annual meeting, and a report is written for the WHO Director General and Executive Board and published thereafter. If specific projects are incomplete, extensions can be requested.

Since the eradication of smallpox, the genome sequences of ~ 50 variola virus isolates have been determined and the sequence deposited in public databases. These sequences have enabled the development of sensitive and specific, polymerase chain reaction-based tests for variola virus. In addition, variola virus neutralizing monoclonal antibodies have been isolated and deployed to develop lateral flow tests for variola virus antigens. Smallpox was a disease restricted to humans and variola virus was able to only infect man or, experimentally, primates. Despite the absence of a satisfactory animal model for smallpox, safer and better characterized vaccines and two smallpox therapeutics (TPOXX and Tembexa) have been approved by regulatory agencies.

In the present study, Hutson and colleagues developed a small animal model for smallpox based on immunodeficient mice that were engrafted with part of the human immune system. In these humanized mice, variola virus can replicate and spread, and although the disease induced is not exactly the same as human smallpox, the model may be useful for testing new therapeutic drugs for smallpox.

Looking forward, the 1996 WHA recommendation that stocks of variola virus should be destroyed has not been revoked. Rather, its implementation has been delayed, and when last debated by WHA additional research was authorized for several more years. However, the ability to derive poxviruses from chemically synthesized genomes may undermine any decision to destroy existing isolates.

